# Health Care Access and Glycemic Control Among U.S. Adults With Diabetes: Analysis of National Health and Nutrition Examination Survey, 2011–2018

**DOI:** 10.7759/cureus.108950

**Published:** 2026-05-16

**Authors:** Nneka Muoghalu, Akinyele Oladimeji, Emeka Okwuokei, Princess Okoronkwo, Emmanuel C Nwaokobia, Theresa M Mambu, Okelue E Okobi

**Affiliations:** 1 Public Health, University of Liverpool, School of Tropical Medicine, Liverpool, GBR; 2 General Medicine, University College Hospital, Ibadan, NGA; 3 Family Medicine, Alberta Health Services, Edmonton, CAN; 4 Internal Medicine, Hywel Dda University Health Board, Aberystwyth, GBR; 5 Pharmaceutical science, Harrisburg University of Science and Technology, Harrisburg, USA; 6 Family Medicine, 168 Medical Group, Weston-super-Mare, GBR; 7 General Medicine, Alexandria Medical Associates, Alexandria, USA; 8 Family Medicine, Larkin Community Hospital Palm Springs Campus, Miami, USA

**Keywords:** diabetes, glycemic control, hba1c, health care access, health disparities, nhanes

## Abstract

Background

Diabetes remains a major public health concern in the United States, with suboptimal glycemic control contributing to increased risk of complications. Access to health care is often considered important for diabetes management, yet its relationship with glycemic outcomes remains inconsistent.

Objective

This study examined the association between health care access and glycemic control among U.S. adults with diabetes using nationally representative data.

Methods

A cross-sectional analysis was conducted using data from the National Health and Nutrition Examination Survey (NHANES) from 2011 to 2018. The study included adults aged 18 years or older with diabetes. Poor glycemic control was defined as glycated haemoglobin (HbA1c) ≥ 7%. Health care access measures included insurance status, usual source of care, and health care visits. Survey-weighted logistic regression was used to estimate adjusted associations while accounting for the complex sampling design.

Results

Health care access variables were not significantly associated with poor glycemic control after adjustment. Male sex and Hispanic ethnicity were associated with higher odds of poor glycemic control. Other demographic and clinical variables showed no significant associations.

Conclusion

This study highlights that health care access alone may not explain differences in glycemic control. Broader factors related to patient characteristics and social conditions should be considered in efforts to improve diabetes outcomes.

## Introduction

Diabetes mellitus is a global public health issue that is particularly prevalent in the United States, where its incidence continues to increase in parallel with rates of obesity, population aging, and sedentary lifestyles [1, 2]. Good control of blood sugar is a major determinant of risk for developing both microvascular and macrovascular problems related to diabetes, such as retinopathy, nephropathy, cardiovascular disease, and premature death [3,4]. The most widely used measure of blood sugar control in people with diabetes over time is the glycated haemoglobin (HbA1c) test, which reflects an individual's long-term glucose level and serves as an essential indicator for the management and prognosis of diabetes [5]. Despite improvements in the treatment of diabetes as well as recommendations from clinical guidelines, a large number of adults diagnosed with diabetes do not reach their optimal blood sugar control, thus indicating continuing gaps in care and management of diabetes [6, 7].

Access to health care services is a key component of the effective management of chronic illnesses like diabetes; it involves multiple aspects, such as insurance coverage, a usual source of care, and the frequency and quality of use of health care services [8]. Individuals who have access to health care services are significantly more likely than those without access to receive timely diagnosis, appropriate medication, regular monitoring, and education on making lifestyle changes, all of which will be necessary to achieve better blood sugar levels [9, 10]. Conversely, limited access to health care services can hinder obtaining a timely diagnosis (delayed), poor adherence (non-compliance) to prescribed medications, and inadequate monitoring, leading to an increased risk of having poor blood sugar control and consequently increased risk for complications associated with poorly controlled diabetes [11].

Access to health care in the U.S. is still unequal depending on social, racial, and geographic factors. Consequently, the way diabetes is treated differs across these groups [12]. Those without insurance or a usual place to receive care face additional barriers to accessing preventive services and ongoing care needed to maintain blood sugar levels [13]. Furthermore, the way people use the health care system, including frequency of physician appointments, who they see (general practitioners or specialists), and how often they receive preventive care, may affect how diabetic patients control their blood sugar levels [14, 15]. Therefore, researchers need to understand how these factors work together, as they will inform health policies and improve population health [16].

While previous research has focused on individual aspects of access to health care or glycemic control, fewer studies have examined multiple aspects of access to health care in relation to one or more aspects of glycemic control using a nationally representative sample [17, 18]. The relationship between access factors and glycemic control when considering all types of access, such as having health insurance, having a usual place, and how health services were used, continues to be unclear [19].

The study employs the National Health and Nutrition Examination Survey (NHANES). For a better opportunity to examine these associations across diverse populations. NHANES provides an opportunity to use both clinical/laboratory data and questionnaire data to assess a patient's health status and other indicators of access to health care [20]. The objective of the study is to evaluate the association between health care access (including insurance coverage, usual source of care, and health care utilization) and glycemic control among U.S. adults with diabetes using NHANES data. The study's findings will contribute to the existing literature on healthcare access and glycemic control among adults with diabetes.

## Materials and methods

Study design and data source

This study was a cross-sectional analysis of data from the National Health and Nutrition Examination Survey, NHANES, covering the 2011 to 2018 cycles [21]. NHANES is a nationally representative survey of the noninstitutionalized United States population that uses a complex, multistage probability sampling design. Data are collected through household interviews, standardized physical examinations, and laboratory testing conducted in mobile examination centers. The present analysis used publicly available data files, including demographic, questionnaire, and laboratory components relevant to diabetes, health care access, and glycemic control.

Study population

The study population consisted of adults aged 18 years or older with diabetes. Diabetes was defined based on self-report of a physician diagnosis or current use of insulin or oral hypoglycemic medication. Participants with missing glycohemoglobin measurements were excluded. Additional exclusions were applied for missing covariate data as part of a complete case analytic approach. The final analytic sample included 2,688 participants, corresponding to a weighted population of 22,535,640 United States adults with diabetes.

Variables and measures

The primary outcome was glycemic control, defined using glycohemoglobin levels measured in the laboratory. Poor glycemic control was defined as HbA1c ≥ 7%, and controlled glycemia was defined as HbA1c < 7%. The primary exposures were measures of health care access, including health insurance coverage, usual source of care, and frequency of health care visits in the past year. Health insurance and usual source of care were analyzed as binary variables. Health care visits were categorized into 0 visits, one to three visits, and four or more visits per year. Covariates included age, sex, race and ethnicity, education level, income to poverty ratio, body mass index, hypertension status, history of heart failure, history of coronary heart disease, and smoking status. Race and ethnicity and education were treated as categorical variables with specified reference categories, and continuous variables were retained in their original scale.

Missing data

Missing data were assessed for all variables included in the analysis. The proportion of missingness was low for most variables, including education, 0.45%, body mass index, 2.20%, hypertension, 0.22%, heart failure, 0.26%, coronary heart disease, 0.26%, smoking status, 0.13%, insurance status, 0.06%, and usual source of care, 0.80%. The income-to-poverty ratio had a higher proportion of missing values, 10.25%. A complete case approach was applied, and participants with missing data on any variable included in the final model were excluded. This method maintained consistency across analyses and ensured that all regression estimates were based on the same analytic sample.

Statistical analysis

All analyses accounted for the complex NHANES survey design, including strata, primary sampling units, and examination weights. For the combined survey cycles, appropriate weighting adjustments were applied to produce nationally representative estimates. Descriptive statistics were generated to summarize participant characteristics by glycemic control status. Group comparisons by the outcome variable were conducted using survey-adjusted t-tests for continuous variables and survey-adjusted chi-square tests for categorical variables. Multivariable logistic regression models were used to estimate adjusted odds ratios and 95% confidence intervals for the association between health care access variables and poor glycemic control. Covariates were selected based on clinical relevance and prior literature. Multicollinearity was assessed using variance inflation factors, and variance inflation factor (VIF) values ranged from 1.04 to 1.53, with a mean VIF of 1.20, indicating no evidence of problematic multicollinearity. All statistical analyses were conducted in Stata version 18 (StataCorp LLC., College Station, Texas) [22].

Ethical considerations

NHANES data are publicly available and deidentified. The survey protocol was approved by the National Center for Health Statistics Research Ethics Review Board, and all participants provided written informed consent at the time of data collection. The present study used secondary deidentified data and did not require additional institutional review board approval.

## Results

Table 1 below presents the baseline characteristics of participants stratified by glycemic control status.

**Table 1:** Characteristics of Participants by Glycemic Control Status Among U.S. Adults with Diabetes (NHANES 2011–2018; Unweighted N = 2,688; Weighted N = 22,535,640) HbA1c, glycated hemoglobin; GED, general educational development. Values are survey-weighted counts and column percentages for categorical variables and weighted mean and standard deviation for continuous variables. Group comparisons were conducted using survey-adjusted t-tests for continuous variables and survey-adjusted chi-square tests with design-based F-statistics for categorical variables. The asterisk (*) indicates statistical significance at p < 0.05. The dash (–) indicates sections that have been intentionally left blank. The table was generated by the authors using Stata version 18 [22].

Characteristic	HbA1c < 7% (Controlled, N = 12,123,672)	HbA1c ≥7% (Poor Control, N = 10,411,967)	Test statistic	p-value
Age (years), mean ± SD	60.30 ± 13.62	59.29 ± 12.92	t = 1.59	0.116
Body mass index (kg/m²), mean ± SD	33.03 ± 7.47	33.73 ± 7.92	t = -1.54	0.129
Income-to-poverty ratio, mean ± SD	2.82 ± 1.58	2.69 ± 1.66	t = 1.13	0.261
Gender, n (%)	–	–	F = 15.50	0.0002*
Male	5,629,324 (46%)	5,810,407 (56%)	–	–
Female	6,494,348 (54%)	4,601,560 (44%)	–	–
Race/Ethnicity, n (%)	–	–	F = 3.39	0.014*
Mexican American	931,557 (8%)	1,198,192 (12%)	–	–
Other Hispanic	620,379 (5%)	710,030 (7%)	–	–
Non-Hispanic White	7,878,674 (65%)	6,055,939 (58%)	–	–
Non-Hispanic Black	1,537,951 (13%)	1,493,276 (14%)	–	–
Non-Hispanic Asian	641,898 (5%)	566,598 (5%)	–	–
Other race	513,213 (4%)	387,932 (4%)	–	–
Education level, n (%)	–	–	F = 1.50	0.229
Less than high school	2,452,985 (20%)	1,977,048 (19%)	–	–
High school/GED	2,889,135 (24%)	2,899,675 (28%)	–	–
College or above	6,781,552 (56%)	5,535,244 (53%)	–	–
Health insurance, n (%)	–	–	F = 7.36	0.009*
Uninsured	942,250 (8%)	1,248,927 (12%)	–	–
Insured	11,181,422 (92%)	9,163,040 (88%)	–	–
Usual source of care, n (%)	–	–	F = 4.35	0.041*
No	501,354 (4%)	665,210 (6%)	–	–
Yes	11,622,318 (96%)	9,746,757 (94%)	–	–
Healthcare visits (past year), n (%)	–	–	F = 0.05	0.880
0 visits	96,690 (1%)	86,818 (1%)	–	–
1–3 visits	1,823,981 (15%)	1,522,231 (15%)	–	–
≥4 visits	10,203,001 (84%)	8,802,918 (85%)	–	–
Hypertension, n (%)	–	–	F = 0.01	0.937
No	3,842,618 (32%)	3,323,520 (32%)	–	–
Yes	8,281,054 (68%)	7,088,447 (68%)	–	–
Heart failure, n (%)	–	–	F = 0.01	0.907
Yes	1,049,708 (9%)	922,045 (9%)	–	–
No	11,073,964 (91%)	9,489,922 (91%)		
Coronary heart disease, n (%)	–	–	F = 1.30	0.258
Yes	1,304,205 (11%)	1,331,502 (13%)	–	–
No	10,819,467 (89%)	9,080,465 (87%)	–	–
Smoking status, n (%)	–	–	F = 0.84	0.364
Non-smoker	6,105,391 (50%)	5,006,252 (48%)	–	–
Smoker	6,018,281 (50%)	5,405,715 (52%)	–	–

The findings from the table above indicate that the mean age was 60.30 ± 13.62 years among participants with controlled glycemia and 59.29 ± 12.92 years among those with poor control, with no statistically significant difference (p = 0.116). Mean body mass index was 33.03 ± 7.47 kg/m² in the controlled group and 33.73 ± 7.92 kg/m² in the poor control group (p = 0.129). The income-to-poverty ratio was similar between groups, 2.82 ± 1.58 and 2.69 ± 1.66, respectively (p = 0.261).

A higher proportion of males was observed in the poor control group, 5,810,407 (56%), compared with 5,629,324 (46%) in the control group, with a statistically significant difference (p = 0.0002). Differences in race and ethnicity were also statistically significant (p = 0.014), with higher proportions of Mexican American participants, 1,198,192 (12%), and other Hispanic participants, 710,030 (7%), in the poor control group compared with the control group, 931,557 (8%) and 620,379 (5%), respectively.

Education level did not differ significantly between groups (p = 0.229). A higher proportion of uninsured individuals was observed in the poor control group, 1,248,927 (12%), compared with 942,250 (8%) in the control group (p = 0.009). Similarly, lack of a usual source of care was more frequent among those with poor control, 665,210 (6%), compared with 501,354 (4%) in the controlled group (p = 0.041). The distribution of healthcare visits was similar between groups (p = 0.880). Hypertension, heart failure, coronary heart disease, and smoking status did not differ significantly between groups (p = 0.937, 0.907, 0.258, and 0.364, respectively).

Table 2 below presents the adjusted associations between health care access variables and poor glycemic control.

**Table 2:** Adjusted Association Between Health Care Access and Poor Glycemic Control (NHANES 2011–2018) CI, confidence interval; GED, general educational development. Models were estimated using survey-weighted logistic regression accounting for strata, clusters, and sampling weights. Reference categories were female sex, non-Hispanic White race, college or higher education, no health insurance, no usual source of care, and zero healthcare visits. The asterisk(*) denotes statistical significance (p < 0.05). The table was generated by the authors using Stata version 18 [22].

Variable	Adjusted Odds Ratio	95% CI	p-value
Age (years)	1.00	0.99–1.01	0.852
Income-to-poverty ratio	0.97	0.88–1.06	0.448
Body mass index (kg/m²)	1.02	1.00–1.04	0.055
Health insurance (Yes vs No)	0.74	0.50–1.10	0.130
Usual source of care (Yes vs No)	0.75	0.45–1.27	0.280
Healthcare visits (1–3 vs 0 visits)	1.34	0.67–2.65	0.400
Healthcare visits (≥4 vs 0 visits)	1.42	0.65–3.13	0.376
Sex (Male vs Female)	1.54	1.25–1.90	< 0.001*
Race/Ethnicity	–	–	–
Mexican American vs Non-Hispanic White	1.71	1.17–2.49	0.006*
Other Hispanic vs Non-Hispanic White	1.61	1.17–2.21	0.004*
Non-Hispanic Black vs Non-Hispanic White	1.32	1.01–1.72	0.040*
Non-Hispanic Asian vs Non-Hispanic White	1.38	0.98–1.94	0.067
Other race vs Non-Hispanic White	0.88	0.50–1.54	0.641
Education level	–	–	–
Less than high school vs College+	0.80	0.60–1.07	0.137
High school/GED vs College+	1.18	0.90–1.56	0.221
Hypertension (Yes vs No)	1.01	0.76–1.33	0.961
Heart failure (Yes vs No)	1.06	0.69–1.62	0.778
Coronary heart disease (Yes vs No)	0.78	0.55–1.09	0.141
Smoking (Yes vs No)	1.05	0.85–1.29	0.675

The results indicate that health insurance coverage was not significantly associated with poor glycemic control after adjustment (aOR = 0.74, 95% CI: 0.50-1.10, p = 0.130). Usual source of care and healthcare visits were also not significantly associated with the outcome. Age and income-to-poverty ratio were not associated with poor glycemic control. Body mass index showed a borderline association (aOR = 1.02, 95% CI: 1.00-1.04, p = 0.055).

Male sex was significantly associated with higher odds of poor glycemic control compared with female sex (aOR = 1.54, 95% CI: 1.25-1.90, p < 0.001). Mexican American participants had higher odds of poor glycemic control compared with non-Hispanic White participants (aOR = 1.71, 95% CI: 1.17-2.49, p = 0.006), and other Hispanic participants also had higher odds (aOR = 1.61, 95% CI: 1.17-2.21, p = 0.004). Non-Hispanic Black participants showed a statistically significant association with increased odds of poor glycemic control (aOR = 1.32, 95% CI: 1.01-1.72, p = 0.040), whereas the association for non-Hispanic Asian participants did not reach statistical significance. Education level, hypertension, heart failure, coronary heart disease, and smoking status were not significantly associated with poor glycemic control.

**Figure 1:**
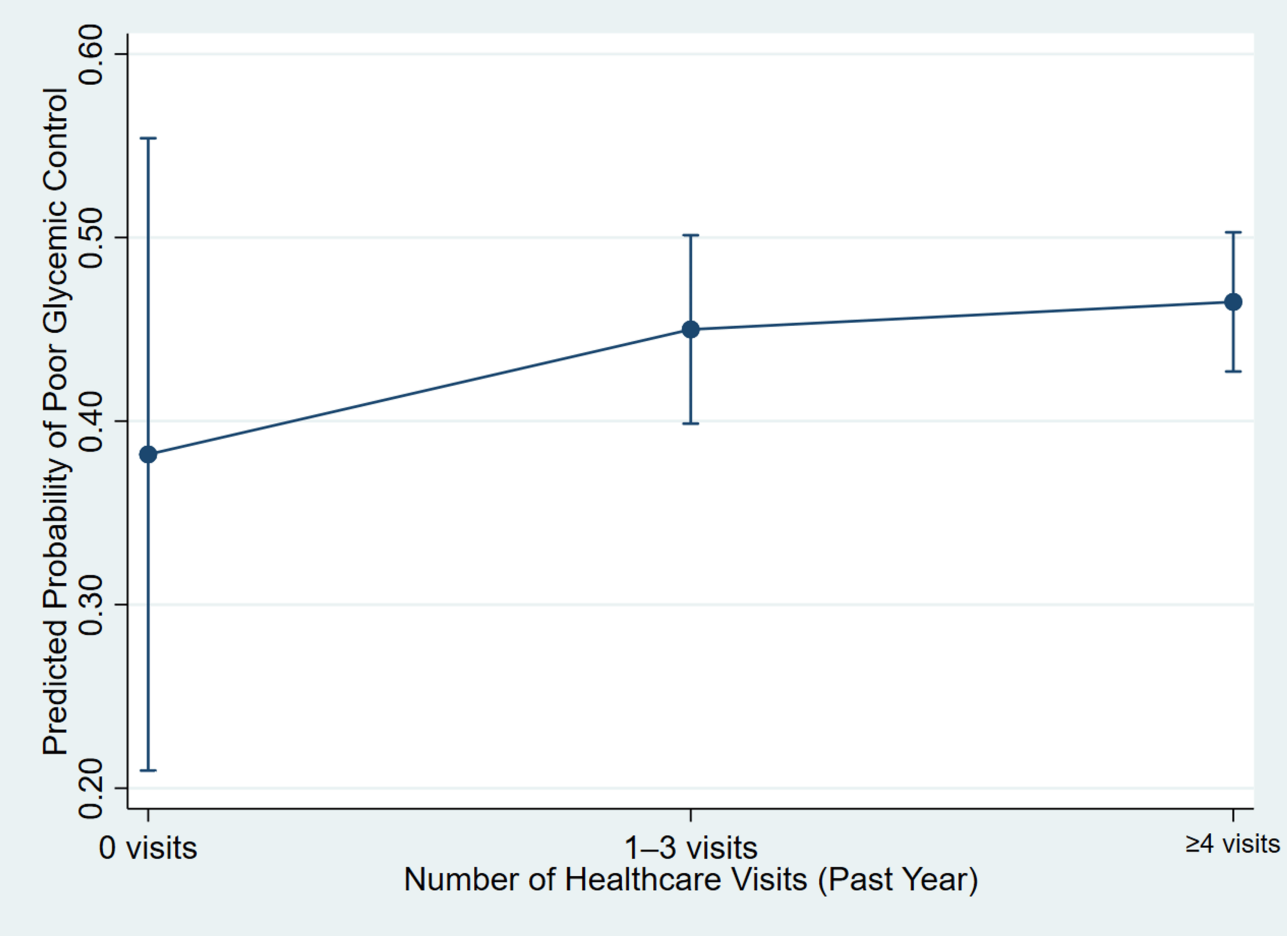
Predicted Probability of Poor Glycemic Control by Healthcare Visits Category Predicted probabilities of poor glycemic control by healthcare utilization category (0 visits, 1–3 visits, ≥4 visits). Estimates are derived from adjusted logistic regression models. Error bars represent 95% confidence intervals.

Figure 1 illustrates the predicted probability of poor glycemic control across categories of healthcare visits.

The figure shows that the predicted probability of poor glycemic control increased from the lowest category of healthcare visits to higher categories. Participants with zero visits had a lower predicted probability compared with those with one to three visits and those with four or more visits. Confidence intervals overlap across categories, indicating that these differences are not statistically significant.

## Discussion

The findings of this study provide insight into the relationship between health care access and glycemic control among U.S. adults with diabetes. The results showed that measures of health care access, including insurance coverage, usual source of care, and frequency of health care visits, were not significantly associated with poor glycemic control after adjustment for demographic and clinical factors.

Although Figure 1 suggests a slight increase in the predicted probability of poor glycemic control with a higher frequency of health care visits, this trend was not statistically significant in the adjusted analysis. This pattern may reflect reverse causation or confounding by disease severity, whereby individuals with poorer glycemic control are more likely to require more frequent health care visits for monitoring and management rather than increased visits leading to worse outcomes.

Biologically, type 2 diabetes is characterized by complex mechanisms including insulin resistance, progressive β-cell dysfunction, and metabolic dysregulation, which collectively drive sustained hyperglycemia and elevated HbA1c levels [3,4]. These intrinsic disease processes are influenced by chronic low-grade inflammation, oxidative stress, and vascular endothelial dysfunction, factors that are not readily modifiable solely through increased health care encounters or insurance coverage [3,4]. Consequently, even with appropriate access, patients may experience persistent hyperglycemia due to the natural progression of the disease. Furthermore, effective glycemic control requires continuous and coordinated management encompassing lifestyle modifications such as dietary optimization, physical activity, and weight management, alongside pharmacologic therapy tailored to individual patient needs [2, 6]. Patient adherence to these recommendations, medication compliance, and sustained engagement in care are critical determinants of outcomes but can be hindered by psychosocial barriers, health literacy limitations, and socioeconomic challenges [6, 7]. These behavioral and social determinants often persist regardless of health care access, highlighting the necessity of comprehensive, patient-centered approaches. In contrast, significant differences were observed by sex and race and ethnicity. Male participants had higher odds of poor glycemic control, and Mexican American and other Hispanic groups also had higher odds compared with non-Hispanic White participants. These findings suggest that disparities in glycemic control persist despite differences in access related measures. Previous studies have reported that gaps in glycemic control remain common even among individuals receiving treatment, indicating that access alone may not be sufficient to achieve optimal outcomes [6,7]. Evidence has also shown that health system factors influence diabetes awareness and management, but improvements in access do not always translate into improved glycemic outcomes [10]. Prior work using NHANES data found that access to care is linked to better management of key diabetes indicators, although this relationship may vary depending on population characteristics and adjustment for confounders [8]. Studies focusing on insurance status have demonstrated mixed findings, with some reporting improved glycemic control among insured individuals and others showing limited differences after adjustment [9,19]. The observed racial and ethnic disparities align with existing literature indicating that minority populations experience differences in diabetes outcomes related to social and structural factors [12,18]. These disparities may reflect differences in disease burden, barriers to effective disease management, and variations in care quality rather than access alone.

Current clinical guidance in the United States emphasizes the importance of achieving and maintaining glycemic targets to reduce the risk of complications. Glycemic control is a central component of diabetes management, as poor control is associated with both microvascular and macrovascular complications [3,4]. National recommendations support regular monitoring of HbA1c levels and individualized treatment approaches to maintain levels below established thresholds. These guidelines also highlight the role of consistent follow-up care and patient engagement in achieving treatment goals. Despite these recommendations, prior studies have shown that a substantial proportion of individuals with diabetes do not meet target HbA1c levels, even among those receiving care [7]. The present findings are consistent with this pattern, indicating that the presence of access-related factors such as insurance and health care utilization does not necessarily ensure adequate glycemic control.

Several mechanisms may help explain the observed associations. Glycemic control depends on a combination of biological, behavioral, and health system factors. While access to care facilitates diagnosis and treatment, effective glycemic control requires sustained adherence to medication, lifestyle modification, and ongoing disease monitoring. Barriers such as treatment adherence, psychosocial factors, and health literacy may influence outcomes independently of access [6,10]. The higher odds of poor glycemic control observed among certain racial and ethnic groups may reflect differences in social determinants of health, including economic conditions, access to healthy foods, and environmental factors that influence disease management [12]. In addition, variations in care delivery, patient engagement, and treatment intensity may contribute to differences in glycemic outcomes. Increased health care utilization does not always indicate effective management, as higher visit frequency may also reflect more severe disease or complications [11,15].

Strengths and limitations of the study

This study has several strengths and limitations. The use of NHANES data allowed for nationally representative estimates and standardized measurement of glycohemoglobin. The analysis accounted for the complex survey design and included a broad set of demographic and clinical covariates, strengthening the validity of the findings.

However, several limitations should be considered. The cross-sectional design limits the ability to assess temporal relationships and precludes causal inference. Several variables were based on self-reports, which may introduce misclassification. Missing data, particularly for the income-to-poverty ratio, resulted in a reduced analytic sample due to the complete case approach, which may introduce selection bias if missingness is not completely random. In addition, residual confounding cannot be excluded, as important determinants of glycemic control, such as medication adherence, diet quality, physical activity, food insecurity, and health literacy, were not available in the dataset. The interpretation of healthcare utilization should also be made with caution, as higher utilization may reflect underlying disease severity rather than improved access, raising the possibility of reverse causation. Furthermore, although covariates were selected based on clinical relevance, the model specification may not fully capture all underlying relationships. Future research should incorporate longitudinal designs, sensitivity analyses, and a broader range of behavioral and social determinants to better understand the pathways influencing glycemic control.

## Conclusions

This study highlights that health care access measures, including insurance coverage, usual source of care, and health care utilization, were not independently associated with glycemic control among U.S. adults with diabetes after accounting for demographic and clinical factors. The findings show persistent differences by sex and race and ethnicity, suggesting that glycemic outcomes are influenced by factors beyond access to care. These results support the need to address broader determinants of diabetes management, including patient engagement, social conditions, and quality of care. Future research should focus on longitudinal designs and incorporate behavioral and social factors such as medication adherence, diet, and health literacy to better understand pathways influencing glycemic control and to inform targeted interventions.

## References

[1] Crawford AG, Cote C, Couto J (2010). Prevalence of obesity, type II diabetes mellitus, hyperlipidemia, and hypertension in the United States: findings from the GE Centricity Electronic Medical Record database. Popul Health Manag.

[2] Geiss LS, Wang J, Cheng YJ (2014). Prevalence and incidence trends for diagnosed diabetes among adults aged 20 to 79 years, United States, 1980-2012. JAMA.

[3] Stolar M (2010). Glycemic control and complications in type 2 diabetes mellitus. Am J Med.

[4] D'Elia JA, Bayliss G, Roshan B, Maski M, Gleason RE, Weinrauch LA (2011). Diabetic microvascular complications: possible targets for improved macrovascular outcomes. Int J Nephrol Renovasc Dis.

[5] Oyeleye-Adegbite OC, Ozojide KO, Akinade O (2025). Association between antidepressant use and hemoglobin A1C levels in adults with comorbid type 2 diabetes mellitus and depression. Cureus.

[6] Blonde L, Aschner P, Bailey C, Ji L, Leiter LA, Matthaei S (2017). Gaps and barriers in the control of blood glucose in people with type 2 diabetes. Diab Vasc Dis Res.

[7] Hankosky ER, Schapiro D, Gunn KB, Lubelczyk EB, Mitroi J, Nelson DR (2023). Gaps remain for achieving HbA1c targets for people with Type 1 or Type 2 diabetes using insulin: results from NHANES 2009-2020. Diabetes Ther.

[8] Zhang X, Bullard KM, Gregg EW (2012). Access to health care and control of ABCs of diabetes. Diabetes Care.

[9] Bolarinwa BO, Ohiri CJ, Sadiq-Onilenla RA, Iziogo SP, Okobi OE (2025). Impact of health insurance status on HbA1c control among United States adults with type 2 diabetes: a cross-sectional analysis of nationally representative data. Cureus.

[10] Ong SE, Koh JJ, Toh SE (2018). Assessing the influence of health systems on Type 2 Diabetes Mellitus awareness, treatment, adherence, and control: a systematic review. PLoS One.

[11] Chan AX, McDermott Iv JJ, Lee TC, Ye GY, Shahrvini B, Radha Saseendrakumar B, Baxter SL (2022). Associations between healthcare utilization and access and diabetic retinopathy complications using All of Us nationwide survey data. PLoS One.

[12] Yedjou CG, Sims JN, Njiki S, Chitoh AM, Joseph M, Cherkos AS, Tchounwou PB (2024). Health and racial disparities in diabetes mellitus prevalence, management, policies, and outcomes in the United States. J Community Med Public Health.

[13] Brown DS, McBride TD (2015). Impact of the Affordable Care Act on access to care for US adults with diabetes, 2011-2012. Prev Chronic Dis.

[14] Quinn CC, Swasey KK, Torain JM, Shardell MD, Terrin ML, Barr EA, Gruber-Baldini AL (2018). An mHealth diabetes intervention for glucose control: health care utilization analysis. JMIR Mhealth Uhealth.

[15] Reynolds EL, Mizokami-Stout K, Putnam NM (2023). Cost and utilization of healthcare services for persons with diabetes. Diabetes Res Clin Pract.

[16] Saulsberry L, Peek M (2019). Financing diabetes care in the U.S. health system: payment innovations for addressing the medical and social determinants of health. Curr Diab Rep.

[17] Alawode O, Humble S, Herrick CJ (2023). Food insecurity, SNAP participation and glycemic control in low-income adults with predominantly type 2 diabetes: a cross-sectional analysis using NHANES 2007-2018 data. BMJ Open Diabetes Res Care.

[18] Laiteerapong N, Fairchild PC, Chou CH, Chin MH, Huang ES (2015). Revisiting disparities in quality of care among US adults with diabetes in the era of individualized care, NHANES 2007-2010. Med Care.

[19] Gold RS, Unkart JT, McClelland RL, Bertoni AG, Allison MA (2021). Health insurance status and type associated with varying levels of glycemic control in the US: the multi-ethnic study of atherosclerosis (MESA). Prim Care Diabetes.

[20] Demmer RT, Zuk AM, Rosenbaum M, Desvarieux M (2013). Prevalence of diagnosed and undiagnosed type 2 diabetes mellitus among US adolescents: results from the continuous NHANES, 1999-2010. Am J Epidemiol.

[21] (2026). National Health and Nutrition Examination Survey (NHANES). https://wwwn.cdc.gov/nchs/nhanes/search/default.aspx.

[22] StataCorp. 2025 StataCorp. 2025.Stata Statistical Software: Release 18. College Station, TX: StataCorp LLC. https://www.stata.com/.

